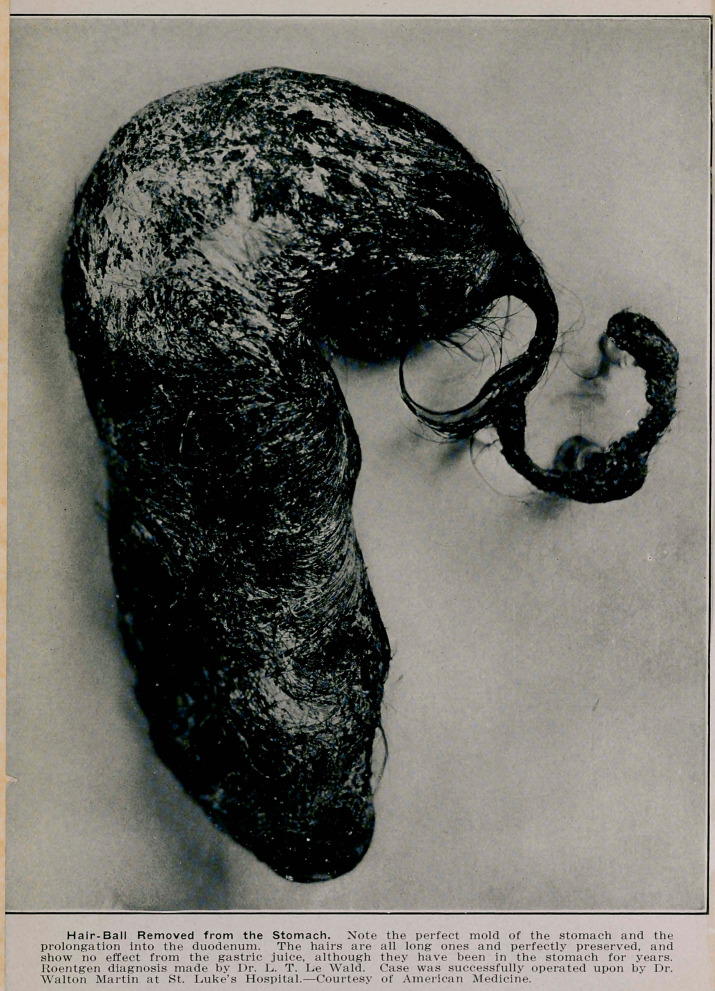# Diagnosis of Cancer of the Colon*Read at the fifteenth annual meeting of the Lake Keuka Surgical and Medical Association, July 9th, 1914.

**Published:** 1914-10

**Authors:** Joseph Burke

**Affiliations:** Attending Surgeon, Sisters’ Hospital, Consulting Surgeon, Emergency Hospital, Buffalo


### BUFFALO MEDICAL JOURNAL

Yearly Volume 70. OCTOBER, 1914 No. 3

### ORIGINAL ARTICLES

The right is reserved to decline papers not dealing with practical medical and surgical subjects, and such as might offend or fail to interest readers. Contributors are solely responsible for opinions, methods of expression and revision of proof.

### Diagnosis of Cancer of the Colon*

By JOSEPH BURKE, Sc. D., M. D.

Attending Surgeon, Sisters' Hospital, Consulting Surgeon, Emergency Hospital, Buffalo.

That a diagnosis of beginning carcinoma of any organ is impossible is incontrovertible; there must be clinical manifestations to enable the surgeon to recognize the existence of that which produces these symptoms. It is possible that in the future when sero-diagnosis shall have reached its perfection that cancer in its very beginning may be accurately determined. The early diagnosis of primary cancer of the liver, spleen, or pancreas is seldom positively made because cancer per se gives rise to no pathognomonic symptoms, its resulting complications being first manifested a long time after the malignant process has begun. On the other hand, carcinoma of the stomach or intestine, depending largely upon its situation and size, is in a great many instances positively ascertained and the result of an early surgical interference life saving to the patient and a credit to the operator. The discussion of the diagnosis of cancer of the colon therefore ought to afford us considerable interest.

**Symptomatology.** The early symptoms of cancer of the colon are for a long time very indistinct and difficult of interpretation, and, apart from the anatomical site of the cancer, depend upon three definite factors: first, stenosis of the bowel; second, accompanying intestinal catarrh, and third, ulceration of the growth of either the mucous membrane or externally into some other organ. This last brings to my

*Read at the fifteenth annual meeting of the Lake Keuka Surgical and Medical Association, July 9th, 1914.

mind a case of carcinoma of the colon, (J. W., aged 55 years), in which the patient's first symptoms were those of colitis and loss of weight and strength.

It was only when distressing bladder disturbances began that he sought medical aid and the true nature of the pathology was suspected; there had occurred an ulceration of the growth from the sigmoid into the bladder with the resulting communication of the interior of the colon with the interior of the bladder. In this case when the patient complained of diarrhoea he affirmed also that the colicky pains, preceding and accompanying the emptying of the bowel, radiated to the anus. Had I intercepted this symptom as Rudolph Schmidt points out, my suspicion of sigmoid cancer would have been immediately aroused. In this instance no tumor could be determined by abdominal palpation for a long time, and rectal examination showed no pathology. It was when severe cystitis developed and gas escaped through the urethra that the correct diagnosis was made.

When a pale patient who has never had any infectious disease, possesses no valular lesions and has enjoyed perfect health up to a certain given moment, particularly as regards his digestion, suddenly, with or without dietary indiscretion, e. g., eating of flatulence-producing vegetables, begins to suffer from colicky pains in his abdomen with rumbling noises, and radiation of the pains toward the anus, accompanied by rectal tenesmus, and either in addition to obstinate constipation or diarrhoea notices a great loss of weight and increasing muscular weakness, cancer of some part of the colon should be immediately suspected; when the stools contain blood, mucus or pus, or all three at one time the further suspicion of cancer is strengthened; and if a mass is found in any part of the abdomen with or without visible peristalsis, the almost positive determination that a cancer is present is made. In this connection I want to emphasize the fact that tumors of the sigmoid are very rarely palpated in the early stages; what we do sometimes feel and interpret as a sigmoid cancer is either chronic inflamed and enlarged colon or scybala above the strictured bowel; that not always when we find a mass in the abdomen does it signify the exact location of the suspected cancer as I demonstrated in a case of another carcinoma of the sigmoid flexure in which a hard mass could be palpated in the left upper quadrant and the diagnosis of cancer at that point was made, but the autopsy revealed a circular scirrhus carcinoma of the sigmoid, and the mass that appeared in the upper portion of the descending colon was hardened faeces. This point is important on account of the surgical attempt to relieve the pathological condition, and the placing of the necessary incision.

"The pains of intestinal cancer we find localized around the umbilicus or spread diffusely in the lower abdomen. In some cases the situation of the colic, the pains being locally limited, corresponds to the seat of the disease, and if diagnosed as if intestinal origin will lead one to think of the local cause which, other things being equal, frequently turns out to be a cancer. These pains very frequently radiate into the back, but continued pains in the back do not occur as frequent findings. These pains while occurring frequently at the height of obstipation,some time occur when there is fairly regular bowel movement. On the other hand, they can be absent with the severest obstipation, not occur at all, or set in late in the disease. The absence of colics, therefore, can never be construed against the diagnosis of a possible carcinoma of the large intestine. In speaking of obstipation due to carcinoma of the colon it is important to bear in mind that it not infrequently shows deceptive remissions. These may be explained by compensatory hypertrophy of the portion of the bowel above the stenosis or to an opening of the passage through ulceration."

Profuse haemorrhage from the bowel is a seldom occurrence in colon carcinoma; when it does take place it generally originates in ulcerating cancer of the sigmoid or rectum and every clinical means should be used to determine its origin. I saw a case in which the first symptom which caused the patient to consult a doctor was an alarming bright red haemorrhage. Two years afterward this patient was suddenly taken with acute abdominal pain and the attending physician diagnosed acute appendicitis and rushed the patient to the hospital for immediate operation. Upon opening the abdomen however, the surgeon found fecal masses free in the abdominal cavity, due to a perforation of the sigmoid resulting from ulcerating cancer; he found the appendix normal.

Tarry stools never occur in carcinoma of the colon.

The copious evacuations which occur in the late stages of cancer of the bowel, are scarcely ever influenced by therapeutic measures directed against chronic intestinal catarrh, such as diet, opium, etc., and this fact may serve as a diagnostic reminder. If a doubt exists as to the anatomical situation of the tumor, the X-ray in recent time has become a valuable adjunct to our diagnostic armamentarium, as a case I will describe here aptly illustrates.

Mr. P. Z., aged forty years; occupation, broom-maker.

Family history—Gives no evidence of malignant disease.

Previous history—Patient affirms that he never suffered from any illness up to the time of the present one. No infections. Present illness; about a year ago patient began to complain of gas and pain in the right side of the abdomen; the exact localization was indefinite, but the pain was referred to the right quadrant. The bowels have been loose and lately there has been a watery diarrhoea, and for this reason as well as for the colicky pains in the abdomen, he seeks the advice of a physician. His appetite is very good. Up to one year ago the patient's digestion was perfect; he never suffered from bowel trouble until then, when he suddenly noticed his diarrhoea. Lately he complains of a feeling of weakness and is easily fatigued upon exertion. He has lost 28 pounds during the past year. He has never passed any blood; the only difference in the alvine discharges was that they had become watery. There has been no nausea, no vomiting.

Status Praesens.—Patient shows a peculiar, almost yellowish, paleness of the skin and mucous membranes. He is emaciated. Conjuntivae not icteric; tongue somewhat coated but moist. There are no palpable lymphatic glands in the neck, nor in any other part of the body. Skin lax shows loss of fat. Lungs and heart normal. Abdomen: the abdomen is flat, no ascites; there is no visible peristalsis. On the right side, just above a line drawn above the umbilicus and below the end of the eleventh rib, is a slight elevation. Palpation reveals this to be hard, somewhat tender mass, which is irregular in outline and movable, but is attached to the lower border of the liver. It is movable during inspiration; it can be held fixed during expiration. Bimanually, ballottement can be distinctly felt. There is no pulsation over the tumor, it cannot be made to disappear under the ribs. There is no rigidity of the right rectus muscle. There can be heard no gurgling, no peritoneal friction sounds over the tumor. Urine normal. Liver and spleen not enlarged. Faeces show no pathological elements, no blood. Blood: secondary anaemia; haemoglobin 75 per cent.

Considering in this case:

(a) sudden disturbances of bowel function, after previous perfect health.

(b) loss of weight, 28 pounds, and increasing muscular weakness.

(c) colicky pains in abdomen. (d) secondary anaemia, and,

(e) tumor in right hypochondrium—the diagnosis of malignant tumor was immediately suspected. The fact that the tumor was hard, tender and irregular, not round and smooth in outline, passively movable and could be held fixed during expiration, excluded gall-bladder in the diagnosis; and, further, the fact that the mass could not be pushed up under the ribs and made to disappear, argued against kidney origin; ballottement characterizes kidney tumor, so does a mass which moves downwards with inspiration, and both of these signs were present in our case. Coupled with this the observation that there was no blood nor mucus nor pus in the stools, no peritoneal friction sounds heard over the tumor, no peristalsis visible, caused us to hesitate a little in our positive declaration that we had to do with hepatic flexure carcinoma. I have two X-ray plates of the colon which I present here; it will be seen how beautifully the differential diagnosis was positively determined. In Fig. 1 will be observed the rectum, sigmoid, descending and transverse colon, and then an abrupt cessation of the shadow, as if the bowel were cut off at the hepatic flexure; it will be noticed where the caecum should be seen are two or three fine shadows, showing a trickling of bismuth through the constricted mass of the flexure. In the second plate (Fig. 2) taken fifteen minutes after the first, will be noticed a little more distinctly, shadows of the bismuth that has passed through after fifteen minutes retroperistalsis. The X-ray, as may be seen, makes very plain that we had to do with constriction of the hepatic flexure of the colon and excludes the kidney. I want to say here that a sometimes very valuable aid in the differential diagnosis of tumor of colon, and kidney, that of artificial inflation of the colon, was not done because we thought it unnecessary and dangerous in this instance.

At operation I found carcinoma of the hepatic flexure. On the twentieth day after the operation, two X-ray plates were made to which I wish to call attention. In this place (Fig. 3) will be seen the bismuth-filled ascending and transverse colons, and some of the bismuth forced through the anastomosis opening into the ileum. In the plate (Fig. 4) taken ten minutes after Fig. 3, the half of the transverse colon nearer the anastomosis is seen contracted and almost empty of bismuth, while the ileum is seen to contain much more bismuth than Fig. 4.

Differential Diagnosis.—The differential diagnosis of cancer of the colon can best be considered by a study of the flexures, situations where the growths most frequently occur.

Beginning at the caecum, we find two pathological conditions that can simulate carcinoma, appendicitis in old people and ileocaecal tuberculosis. There are cases in which the differential diagnosis between caecum carcinoma and appendicitis in the beginning gives rise to great speculation, when there exist elevation of temperature and sometimes repeated chills, as well as acute local pain. But here, as well as in all cases, the taking of a very careful previous history up to the time, and exact detailed symptomatology of the present illness, ought to be of great diagnostic aid. Some observers have claimed that temperature in itself speaks against carcinoma, but in this they absolutely err, because temperature elevation is not a seldom phenomenon in gastro-intestinal cancer, as Freuweiler, of Zurich, conclusively demonstrated. Fromme, of Halle, claims that this fever in cancer is due to destruction of the primary tumor, large lymph-channels being opened up, and a great amount of bacteria brought to the lymph-glands and their toxins permeating the blood. Hence I would suggest in the differentiation of bowel carcinoma from appendicitis in elderly people, that we pay absolutely no attention to the temperature as against carcinoma, but rather depend more upon the previous history of the patient. However, both conditions demand early surgical attack, and one who opens the abdomen to operate an appendix ought to be ready to do a radical operation in case his pathology proves to be a cancer. Yet a perfect diagnosis of carcinoma would permit a few days preparation and in my opinion old people can stand a few days toning up. Tuffier has reported cases of malignant diseases of the caecum with abscess formation. As an example of caecum carcinoma in which fever is present as a prominent symptom, I will give the history of a case in detail. On account of the suddenness of the onset of abdominal pain, temperature 103, pulse 105, and exquisite tenderness on palpation, as well as a defined mass in the right iliac region, a diagnosis of acute appendicitis with perityphlitis was suspected by another physician who saw the patient on the second day of the acute attack. I saw the patient on the twelfth day, and after going into the history of the case minutely, obtained the following data:

Female, fifty years of age, married, one child twenty-two years of age. Father died at seventy-eight with paralysis. Mother at sixty-two; cause indefinite. Patient as child never had any infectious diseases until she was fourteen years of age, when she suffered from double quotidian malaria. Last menstruation five months ago.

Present illness—Up to one year ago she was perfectly well and able to do her household work, including washing, without fatigue. At that time and up to six months ago she noticed that she was losing weight and was easily fatigued in doing her work, and had to give it up. Her daughter noticed that her complexion was becoming paler and that she looked bad. About six months ago she noticed that unless she took a physic (Cascara) every day her bowels would not move. This obstinate constipation has persisted up to the present time; there has been no diarrhoea. She has never noticed blood, nor mucus, nor pus in the stool. She has complained of pain when about to urinate and griping pains just before the bowels act. She has never vomited, but lately her appetite has been poor. Twelve days ago she began to complain suddenly of cramps in her right iliac region, which I have already described.

Status Praesens.—The patient has a yellowish, pale, anaemic color. The mucous membrane is pale; there is no jaundice of the conjunctivae; no glands palpable in any part of the body. Tongue clean and moist, heart and lungs normal.

Abdomen: Liver and spleen not enlarged. Inspection of the abdomen shows a rounded prominence, the center of which corresponds to a point an inch below McBurney's point. There are no visible peristaltic waves; palpation reveals this swelling to be hard, exquisitely tender, somewhat uneven mass, which can be slightly moved from side to side. The mass extends about a finger-breadth above a line drawn through the umbilicus, and extends to the left about an inch beyond the middle line. It measures in its longitudinal diameter five inches, in its transverse diameter 4½ inches. Over the center of this tumor there is a dull tympanitic note. The skin and abdominal wall are movable over the tumor, particularly near the anterior superior spine. There is no rigidity of the right rectus muscle.

October 5: Temperature, 99; pulse, 118; respiration, 20. There is less pain complained of. Left lateral position causes dragging pain referred to the site of the swelling in the right iliac region. The mass is not so tender to palpation, and its limits are more confined than five days ago. It extends to a line drawn through the umbilicus above and not quite to the middle line laterally. The tumor is not respiratory movable. There is no rigidity of the right rectus muscle. The skin and underlying structures comprising the abdominal wall over the site of the swelling can be moved as if separated from and gliding over the tumor. Blood shows the following: Haemoglobin, 75 per cent.; white cells, 10,900; polymorphonuclears, 81 per cent.; small, 6 per cent.; erythrocytes, 3,500,000. Stools contain no blood, no mucus, no pus. Urine negative, Diazo reaction not present.

Considering in this case (1) no previous infections, excepting malaria; (2) loss of weight, 29 pounds in six months; (3) obstinate constipation; (4) increasing muscular weakness; (5) griping pains just before defecation; (6) the absence of vomiting, especially at the onset of and during recent febrile illness; (7) yellowish pale color of skin; (8) the fact that the tumor persisted even after the cessation of the fever; (9) the clean moist tongue, which personally I have never seen in any acute abscess case; (10) and this is the most important point and the one upon which I place the greatest dependence, and which, as far as I am aware, has not had attention called to it in the literature, namely, the fact that the skin and underlying structures of the abdominal wall over the site of the swelling could be moved by the palpating hand as if separated from, and gliding over the tumor. I have never observed a case of abscess in the abdomen large enough to see the outline where the muscles over it were not on guard and which could be moved as above described. I did not therefore hesitate to affirm that we had to do with, not appendicitis with abscess, but carcinoma of the caecum, a diagnosis, the correctness of which was demonstrated at operation. Fever and 81 per cent. polymorphonuclear leucocytes in a count of 10,900 and still no acute abscess are observations to be considered in a negative way.

In differentiating cancer from tuberculosis of the caecum, however, most careful examination of both lung apices for healed tubercular processes, the presence of Diazo reaction, the finding of tubercule bacilli in the stool, and lastly the positive Von Pirquet reaction, should guide the surgeon in the right direction. There are cases of ileocaecal tuberculosis that even after removal cannot be differentiated from cancer except by careful microscopical inspection.

Some cases of recurrent appendicitis with hard, indurated palpable tumor mass, without temperature elevation, with little increase in pulse rate which not infrequently occurs, is another source of worry to the diagnostician and more so to the operator, as was illustrated in a recent case of a man 45 years of age, who had no temperature elevation, pulse less than 90, visible intestinal peristalsis and a hard irregular, tender mass in the right iliac region and symptoms of obstinate constipation approaching complete obstruction. Upon opening the abdomen I found the caecum and ascending colon hard and indurated. The appendix adherent to the caecum and running laterally and posteriorly. The mass was so large that the appendix only was removed because it showed signs of recent acute inflammation. The question of resection was raised but the patient's condition was so bad on the table that it was decided not to do anything further at the time. The patient made an uninterrupted recovery. The mass which was undoubtedly inflammatory disappeared and the patient today is in perfect health.

The differential diagnosis of hepatic flexure carcinoma we have already discussed in our case report; the chief causes of error are gall-bladder and liver neoplasms, and kidney tumors.

I saw a case (Mr. C. W., sixty-three years old), however, that on account of a hard painful mass at the hepatic flexure, the thought of carcinoma of that part was taken into consideration, because the patient had lost considerable weight within a short period, was obstinately constipated, and complained of colicky pains and gas in the abdomen; but from the fact that he was jaundiced somewhat, that the tumor could not be separated from the liver and was not movable, that years before he had had an attack of what I judge was acute biliary colic, I made a diagnosis of gall-stones with Riedel's lobe; and the mass which I misinterpreted was omentum which was infiltrated, attached to and enveloped the gall-bladder and the lower border of the liver.

Carcinoma of the transverse colon is so exceedingly rare that confusion with carcinoma of the greater curvature is of seldom occurrence. The gastric symptoms, such as coffee grounds vomit, the Boas-Oppler bacilli, and the absence of free HCL, and the presence of lactic acid in the stomach contents, need only be mentioned to differentiate it from stomach cancer; then too, the X-Ray ought to be an aid, as a case I shall briefly mention will illustrate.

A patient (Mrs. M., forty-five years of age) complained of great loss of weight and rapidly diminishing strength, and a somewhat painful lump about the size of a lemon which ap-peared in the middle line of the abdomen just above the umbilicus. She complained of no gastric symptoms. On account of the mobility of the tumor mass and the constipation which was present, the possibility that a transverse colon carcinoma existed was thought of. The X-Ray pictures showed definitely a greater curvature carcinoma and normal colon.

There are cases of carcinoma of the transverse colon that rupture into the stomach and give rise to faecal vomiting; the diagnosis of such a condition presents a problem for the surgeon as to operative interference, as the following case will illustrate.

Female, 38 years of age, who had been ill one month with colicky pains and vomiting; suddenly the bowels became obstinately constipated, and there occurred symptoms of total obstruction, that is, the patient vomited formed faecal matter; there was very little distention of the abdomen, there was no visible peristalsis. A tender, hard movable mass about the size of a hen's egg was found with its center about at the umbilicus. Repeated lavage of the stomach always brought formed faecal masses. The pulse averaged eighty to ninety, there was no temperature elevation, the abdomen was soft. The question of diagnosis arose between that of a pure intestinal obstruction and that of either a carcinoma of the stomach communicating with the transverse colon, or of carcinoma of the transverse colon ulcerated into the stomach. The diagnosis of cancer of the transverse colon was made and an exploratory operation was done, under novocaine. I found the mass involving the transverse colon and the posterior wall of the stomach. The glands involving the transverse mesocolon were enlarged and very hard, the diagnosis being carcinoma of the transverse colon communicating with the stomach through its posterior wall. I did not attempt to remove the mass but did a palliative operation, an anastomosis between the caecum and the sigmoid flexure of the colon. I have been astonished at the improvement this patient has made since the operation twelve weeks ago. She has not vomited once, and the bowels have moved freely every day. The patient eats well, sleeps well, and apparently is in very good condition. I am wondering whether the diagnosis of cancer of the transverse colon was correct. The mass is still present and time alone will reveal the true nature of the pathological process.

These X-Ray plates were made twelve weeks after operation; the first (Fig. 5) shows an absence of shadow in the central portion of the transverse colon corresponding to the carcinoma; the entero-anastomosis (caecum and sigmoid) can also be seen.

The second (Fig. 6) and third plates (Fig. 7, made 6½ hours after Fig. 6) show a large stomach whose greater curvature is about at the umbilicus; there is a circumscribed absence of shadow and this corresponds to the above mentioned absence of shadow in the transverse colon. There can be seen shadows of the bismuth leaving the stomach at this point, undoubtedly marking the site of the communication between the stomach and colon. I ascribe two reasons why the faecal current does not flow from the colon into the stomach: first, the entero-anastomosis, second, the peristaltic action of the stomach is far stronger than that of the bowel, especially of the portion that is carcinomatous.

The fourth (Fig. 8) and fifth plates (Fig. 9) afford much speculation, but I think we can interpret them. We can positively identify the stomach overlying the transverse colon, at the prevously described points of absence of shadows in the stomach and transverse colon, the indentation of the stomach corresponding to the absence of shadow in the colon. We see also the bismuth passing from the caecum to the sigmoid through the entero-anastomosis.

Carcinoma of the splenic flexure I have never personally observed, but Schmidt says the only conditions that can be mistaken for it are carcinoma of the stomach and of the spleen. Here again X-Ray should prove of great assistance.

In malignant diseases of the sigmoid where the early pains are referred to the bladder and the left testicle, the error of confounding it with nephrolithiasis can obviously be made; but the absence of pathological urinary changes, blood, pus, etc., the negative X-Ray findings as regards stone in the kidney or ureter, would exclude kidney colic at once.

When we consider the differential diagnosis between carcinoma of the sigmoid and diverticulitis we are up against a very difficult problem, in many cases one resembles the other macroscopically so much, that even at operation the surgeon cannot always positively determine the pathological condition present and the microscope alone decides it. We will consider nevertheless, several clinical features that may lead us out of the wilderness of uncertainty. In sigmoid diverticulitis that is active, there occurs always a palpable mass; not so in cancer; in the latter case there occurs mostly constriction, not palpable. A mass therefore, that appears suddenly in a patient who has complained for a long time of pain and tenderness, especially occurring in attacks, speaks for an infllammatory character of the process and against carcinoma; if the mass disappears and after a time returns, an inflammatory process is almost positive. In cancer there is secondary anaemia and great loss of weight and strength; in most cases of diverticulitis, the patients have been well nourished, of good color and sound musculature, and the weight loss very slight. While increase in temperature and leucocytosis cannot be argued positively against cancer, yet they would favor a diagnosis of inflammation. Macroscopic blood in the stool argues for carcinoma and against diverticulitis; 75 per cent of sigmoid carcinomata have blood in the stools.

We must remember that diverticulitis and carcinoma can co-exist in the same case.

Recently I operated a case of cancer of the sigmoid in a girl 24 years of age. The clinical symptoms were vague, constipation being the prevailing one. One week before I saw her she had impending complete obstruction of the bowels which finally was relieved by repeated enemas. At that time the patient noticed a lare swelling in the left iliac region which disappeared after the bowels began to move. The diagnosis of partial obstruction was made and at operation the cause was found to be colloid adeno-carcinoma of the sigmoid flexure and not diverticulitis. I resected the bowel and made an end-to-end anastomosis. The patient recently left the hospital. She reports a gain of seven pounds in three weeks.

In conclusion I wish to express my thanks to Dr. Leonard Reu, for the excellent X-Ray records.

(See photo-plates on Pages 154-159)

---

**Solidified Alcohol.** Pop. Elect. and Mod. Mechanics, August, 1914. Heat 500 parts of 90% denatured alcohol over a water bath to 140 degrees F. Add 1 part gum lac and 15 parts of dry, shaved Venetian soap, stirring to hasten the mixture and reduce evaporation. Pour while hot into small metal cans and cover tightly. On cooling, the mixture "sets." It can be used as fuel for an alcohol stove, etc., in the original receptacle and extinguished by replacing the cover. The residue after the alcohol has been burned out may be used over again.

## Figures and Tables

**Fig.I. Fig. II. Fig. III. Fig. IV. f1:**
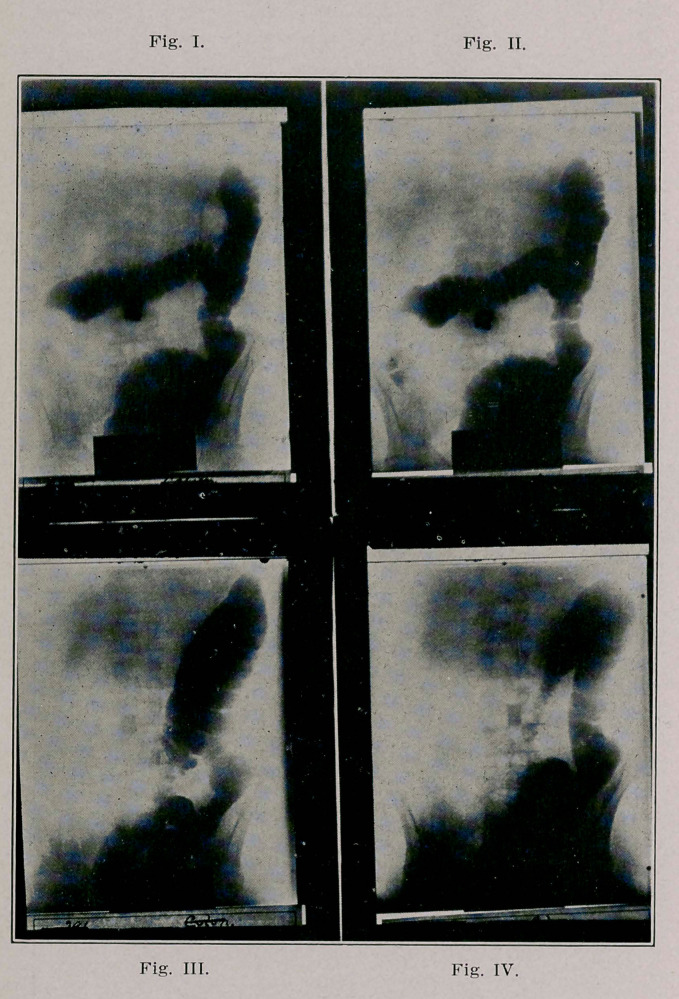


**Fig. V. f2:**
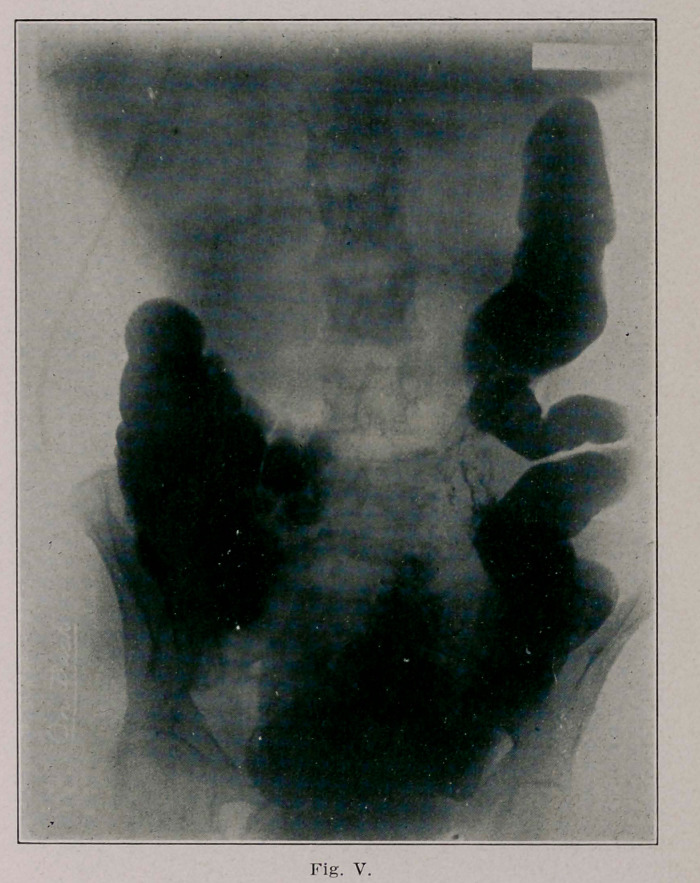


**Fig. VI. f3:**
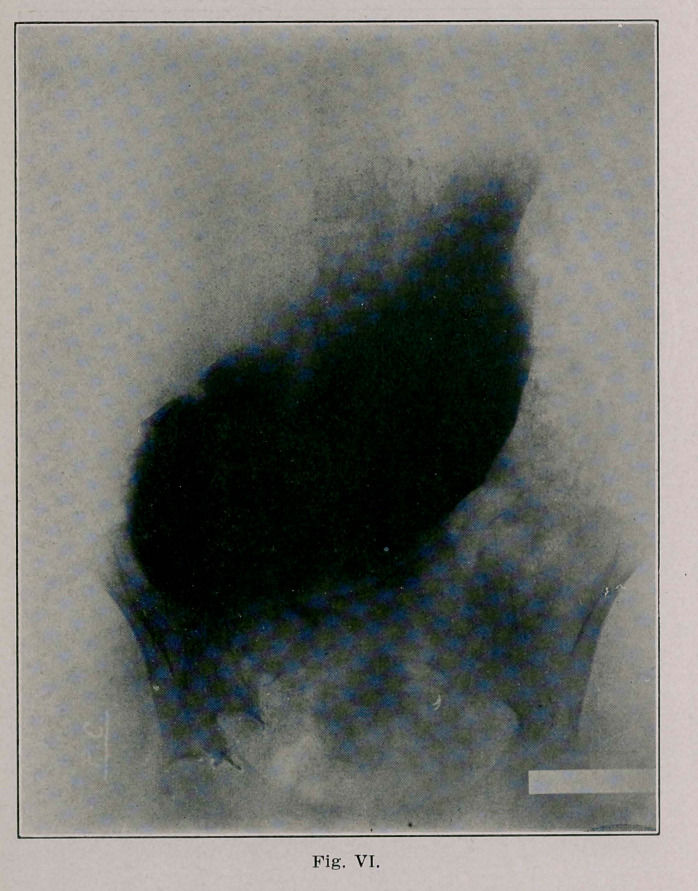


**Fig. VII. f4:**
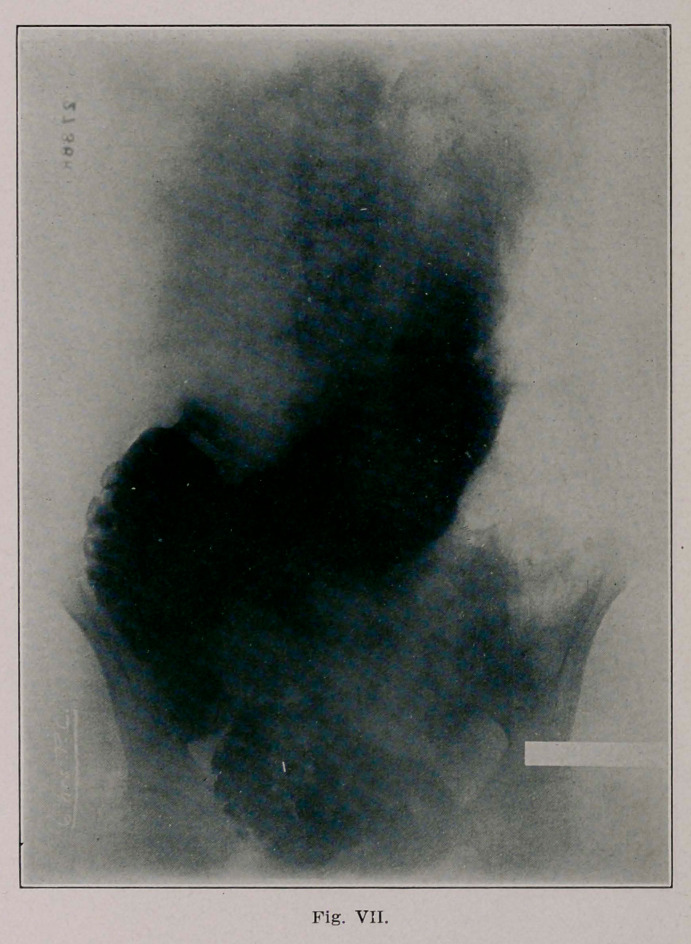


**Fig. VIII. f5:**
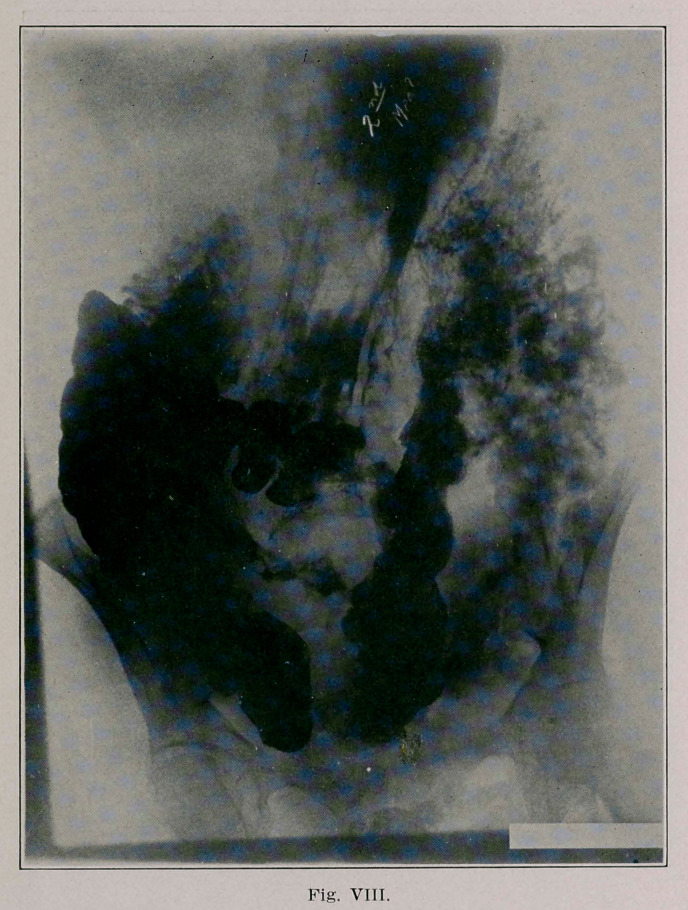


**Fig. IX. f6:**
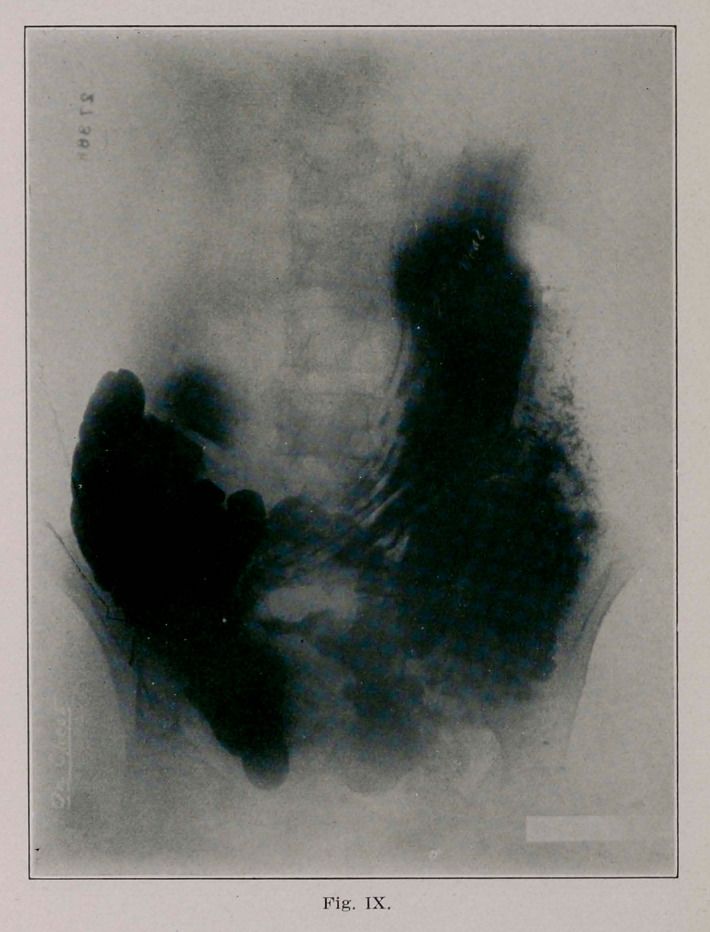


**Figure f7:**